# Self-discharge during treatment for acute recreational drug toxicity: an observational study from emergency departments in seven European countries

**DOI:** 10.1186/s12245-023-00566-1

**Published:** 2023-11-29

**Authors:** Odd Martin Vallersnes, Alison M. Dines, David M. Wood, Fridtjof Heyerdahl, Knut Erik Hovda, Christopher Yates, Isabelle Giraudon, Blazena Caganova, Alessandro Ceschi, Miguel Galicia, Evangelia Liakoni, Matthias E. Liechti, Òscar Miró, Roberta Noseda, Per Sverre Persett, Kristiina Põld, Yasmin Schmid, Irene Scholz, Federico Vigorita, Paul I. Dargan

**Affiliations:** 1 Department of General Practice, University of Oslo, PB 1130, Blindern, Oslo, 0318 Norway; 2 Oslo Accident and Emergency Outpatient Clinic, City of Oslo Health Agency, Oslo, Norway; 3 Clinical Toxicology, Guy’s and St Thomas’ NHS Foundation Trust and King’s Health Partners, London, UK; 4 Clinical Toxicology, Faculty of Life Sciences and Medicine, King’s College London, London, UK; 5 Prehospital Division, Oslo University Hospital, Oslo, Norway; 6 The Norwegian Air Ambulance Foundation, Oslo, Norway; 7 Institute of Clinical Medicine, University of Oslo, Oslo, Norway; 8 Department of Acute Medicine, Oslo University Hospital, Oslo, Norway; 9 Emergency Department and Clinical Toxicology Unit, Hospital Universitari Son Espases, Palma, Spain; 10 European Monitoring Centre for Drugs and Drug Addiction (EMCDDA), Lisbon, Portugal; 11 National Toxicological Information Centre, University Hospital, Bratislava, Slovakia; 12 Division of Clinical Pharmacology and Toxicology, Institute of Pharmacological Sciences of Southern Switzerland, Ente Ospedaliero Cantonale, Lugano, Switzerland; 13 Emergency Department, Hospital Clinic, University of Barcelona, Barcelona, Spain; 14 Clinical Pharmacology and Toxicology, Department of General Internal Medicine, Inselspital, Bern University Hospital, University of Bern, Bern, Switzerland; 15 Clinical Pharmacology and Toxicology, University Hospital and University of Basel, Basel, Switzerland; 16 Emergeny Medicine Department, North-Estonia Medical Centre, Tallinn, Estonia; 17 San Gerardo Hospital [U.O.S. Pronto Soccorso], ASST, Monza, Italy

**Keywords:** Recreational drug toxicity, Self-discharge, Leaving against medical advice, Discharge against medical advice, Leaving without being seen, Substance use, Substances of abuse, Opioids, Poisoning, Intoxication

## Abstract

**Background:**

Self-discharge is a risk factor for readmission and excess mortality. We assess the rate of self-discharge from the emergency department (ED) among presentations for acute recreational drug toxicity and identify factors associated with self-discharge.

**Methods:**

From the Euro-DEN Plus database of presentations to the ED with acute recreational drug toxicity, we extracted data from 11 centres in seven European countries from 2014 to 2017. Self-discharge was defined as taking one’s own discharge or escaping from the ED before being medically cleared. We used multiple logistic regression analyses to look for factors associated with self-discharge.

**Results:**

Among 15,135 included presentations, 1807 (11.9%) self-discharged. Self-discharge rates varied from 1.7 to 17.1% between centres. Synthetic cannabinoids were associated with self-discharge, adjusted odds ratio 1.44 (95% confidence interval 1.10–1.89), as were heroin, 1.44 (1.26–1.64), agitation, 1.27 (1.10–1.46), and naloxone treatment, 1.27 (1.07–1.51), while sedation protected from self-discharge, 0.38 (0.30–0.48).

**Conclusion:**

One in eight presentations self-discharged. There was a large variation in self-discharge rates across the participating centres, possibly partly reflecting different discharge procedures and practices. Measures to improve the management of agitation and cautious administration of naloxone to avoid opioid withdrawal symptoms may be approaches worth exploring to reduce self-discharge.

## Introduction

Self-discharge, usually defined as ‘leaving hospital against medical advice or escaping from the hospital premises’, is associated with an increased risk of readmission and excess mortality [1–3].

Self-discharge is frequent among patients using alcohol or illicit drugs [2, 4]. While self-discharge rates of 1–3% are reported in general hospital populations [1–3, 5], 10–12% of patients treated for alcohol and substance-related disorders self-discharged in a US nationwide register study [5]. Among patients treated for self-harm in UK general hospitals, using alcohol or illegal drugs increased the risk of self-discharge by 49% [6]. Self-discharge occurred nearly twice as often when alcohol was involved among injury patients presenting to Korean emergency departments (EDs) [7]. In a Canadian study, 55% of patients self-discharging from the ED had a substance abuse diagnosis, compared to 9% among other ED patients [8]. Among patients with acute poisoning related to substance use, self-discharge rates of 15–19% are reported [9–11], higher than the 6–11% reported in studies of acute poisoning in general [12–14].

Acute poisoning related to substance use is a marker of hazardous substance use and associated with excess mortality [15, 16]. In a Canadian study, patients treated for illegal drug overdose had ten times more ED presentations than matched controls in the year prior to the overdose and a 19% self-discharge rate compared to 4% [11]. Another Canadian study found that frequent ED visits for substance use disorders were associated with increased mortality in the two following years, and mortality increased steadily with the number of visits [17]. In Norwegian studies, 9% of patients treated for poisoning by substances of abuse represented within a week [18], 30% represented within a year [19], and self-discharge tripled the risk of representation [10]. In the latter study, self-discharging patients were older and more frequently homeless compared to patients not self-discharging, and the poisoning more frequently involved opioids or benzodiazepines [10]. This is the only study we are aware of describing patients self-discharging during treatment for substance use-related poisoning in any detail. These patients constitute an at-risk group in an at-risk situation, and there is a need for further descriptive studies from larger populations and multiple centres. Identifying risk factors for self-discharge may alert clinicians to this vulnerable patient group and might form the basis for tailoring interventions.

### Objectives

We assess the rate of self-discharge from the ED among presentations with acute recreational drug toxicity, compare self-discharge cases to cases not self-discharging, and identify factors associated with self-discharge.

## Methods

### Design

Observational study using data from the European Drug Emergencies Network (Euro-DEN) Plus project on ED presentations with acute recreational drug toxicity. The Euro-DEN Plus methodology has previously been described in detail [9, 20].

### Settings

For this study, we extracted Euro-DEN Plus data for all presentations to 11 centres in seven European countries from 2014 to 2017 (Table 1).

**Table 1:** Presentations with acute recreational drug toxicity 2014–2017: self-discharge rates per participating centre

**Euro-DEN Plus centre**	**Inclusion period**	**Total ED presentations mean per annum**	**Included presentations mean per annum**	**Centre total included presentations** ***n***	**Self-discharge** ***n***	**Other discharge** ***n***	**Self-discharge rate (% of centre total included presentations)**
Oslo OAEOC, Norway^a^	2014–2017	169,901	1500	6001	1026	4975	17.1
Monza, Italy	2017	105,388	113	113	14	99	12.4
London STH, UK	2014–2017	144,030	964	3855	425	3430	11.0
Basel, Switzerland	2014–2017	51,218	238	953	93	860	9.8
London KCH, UK	2014–2017	139,876	414	1657	160	1497	9.7
Bratislava, Slovakia^b^	2016–2017	36,626	74	147	12	135	8.2
Bern, Switzerland	2016–2017	45,077	233	465	26	439	5.6
Lugano, Switzerland^c^	2017, 2nd half	114,178	210	105	4	101	3.8
Oslo OUH, Norway	2014–2017	25,746	191	762	22	740	2.9
Barcelona, Spain	2014–2017	91,742	165	661	18	643	2.7
Tallinn, Estonia	2014–2017	79,727	104	416	7	409	1.7
Total				15 135	1807	13 328	11.9

*ED* Emergency department, *KCH* King’s College Hospital, *OAEOC* Oslo Accident and Emergency Outpatient Clinic, *OUH* Oslo University Hospital, *STH* St Thomas’ Hospital

All centres are single hospital EDs, except:

^a^The OAEOC is a primary care emergency outpatient clinic with limited diagnostic and treatment resources serving as a pre-ED for the four hospitals in Oslo (amongst which the OUH is one). In Norway, patients have to be assessed in primary care or by the ambulance service before going to hospital

^b^The Bratislava centre is the Slovakian National Toxicological Information Centre, which belongs to the University Hospital Bratislava. The centre collects data from three EDs which are part of the University Hospital Bratislava

^c^The Lugano centre is a specialised clinical pharmacology and toxicology unit providing consultancy to a network of four teaching hospitals in southern Switzerland and collects data from the EDs of these four hospitals

### Participants

Patients presenting to a Euro-DEN Plus centre with symptoms and/or signs consistent with acute recreational drug toxicity and/or directly related to recreational drug use were included. Recreational drugs were defined as “any psychoactive compound taken for recreational purposes”. Presentations due to isolated alcohol intoxication or resulting from deliberate self-harm or suicidal attempts were not included. Each centre was responsible for identifying and including all relevant cases, mostly done through a retrospective review of ED patient registration lists and subsequent review of the medical records of potentially eligible cases. We only registered presentations and not individual patients. Accordingly, we use the terms presentations or cases rather than patients when presenting the results.

### Data classification

The Euro-DEN Plus dataset was registered from the patient’s medical record and entered into a purpose-designed Excel spreadsheet in each centre and then returned to the coordinating centre for collation. For this study, we extracted data on age, sex, whether the patient was brought by ambulance, drugs taken, clinical features (respiratory rate at presentation, heart rate at presentation, hyperthermia (temperature ≥ 39.0 °C), hypertension (systolic blood pressure ≥ 180 mmHg, hypotension (systolic blood pressure ≤ 90 mmHg), arrhythmias (any significant arrhythmia other than sinus tachycardia or sinus bradycardia), palpitations, chest pain, vomiting, headache, psychosis (any episode of delusions accompanied by confusion, hallucinations, and lack of insight; as assessed by the treating doctor and documented in the medical records), hallucinations (any false or altered perceptions; visual, auditory, tactile, olfactory, or gustatory), agitation (any episode or threat of disruptive behaviour, violence, or a hostile lack of co-operation), anxiety (any feelings of fear, apprehension, or dread, or note of anxiety in the medical records), seizures (any type of generalised tonic-clonic, myoclonic, partial, or focal seizure that occurs once or more), cerebellar features (positive cerebellar signs on examination, e.g., ataxia, dysdiadochokinesis/dysmetria), lowest conscious level measured by Glasgow Coma Scale (GCS)), treatment given, length of stay, day/time of discharge, and disposition from the ED.

Self-discharge was defined as taking one’s own discharge or escaping from the ED before being medically cleared.

The identification of drug(s) taken was based on the patient’s self-report, the report from any companions, and/or the clinical assessment of the doctor treating the patient as noted in the medical records. 

### Outcome measures

We calculated self-discharge rates as the proportion of presentations ending with self-discharge from the total number of presentations with recreational drug toxicity. Furthermore, we compared demographics, treatment, and time of discharge between self-discharge cases and cases not self-discharging from the ED. We used multiple logistic regression analyses to look for drugs, clinical features, and treatment associated with self-discharge.

### Statistical analyses

Analyses were done in IBM SPSS versions 25–27. For comparisons we used Pearson’s chi-square test for proportions and Mann-Whitney *U* test for continuous variables. When generating the categoric variables bradypnoea (respiratory rate < 12/min), tachypnoea (respiratory rate > 20/min), bradycardia (heart rate < 50/min), and tachycardia (heart rate ≥ 100/min) from the continuous variables respiratory rate and heart rate, missing values were treated as the relevant clinical feature not being present. When categorising the lowest conscious level measured by GCS into alert (GCS 15), drowsy (GCS 9–14), and coma (GCS 3–8), missing values (nearly all missing due to this variable not being collected during the first nine months of the study) were treated as missing and not included in the analyses.

We did separate multiple logistic regression analyses for associations between self-discharge and drugs taken, and between self-discharge and clinical features. Separate analyses were chosen as drugs taken are closely related to which clinical features will be manifest. Hence, we considered these analyses to be two different perspectives on the material. In the drug regression analysis, we included all drugs in the model. The reference group for each drug was not having taken the drug. In the clinical features regression analysis, we also included age, sex, and treatment with naloxone, flumazenil, and sedatives. Age was categorised as ≤ 19 years, 20–39 years, and ≥ 40 years. Reference groups were the clinical feature not being present, GCS 15, male sex, age 20–39 years, or the treatment not given. Furthermore, in the clinical features multivariate regression analysis, we first only included age and sex and the variables significantly associated (*p* < 0.05) with self-discharge in the univariate analyses. We then included each of the remaining variables one by one and discarded them again if not significant. Thus, agitation and naloxone treatment were found to be significantly associated with self-discharge in the multivariate analysis though not in the univariate. Through checking agitation and naloxone treatment with each included variable one by one, we identified sedation treatment as the suppressor variable for agitation, and lowest conscious level as the suppressor variable for naloxone treatment.

## Results

Among the 15,135 presentations to the 11 centres from the Euro-DEN Plus database from 2014 to 2017, 1807 (11.9%) self-discharged, 9188 (60.7%) were medically discharged from the ED, 1157 (7.6%) were admitted to a critical care unit, 548 (3.6%) to a psychiatric ward, 2415 (16.0%) to other hospital wards, 16 (0.1%) died, and in 4 cases disposition was not recorded. The median age was 32 years (IQR 25–40), and 11,738 (77.6%) were male. More than one toxic agent had been taken in 9796 (64.7%) cases. The self-discharge rate ranged from 1.7 to 17.1% between centres (Table 1).

Self-discharge cases had less frequently been sedated compared to other cases, 6.6 vs. 19.8% (*p* < 0.001), and were less frequently given any treatment beyond mere observation, 33.5 vs. 53.5% (*p* < 0.001) (Table 2). Median stay was 2 h 20 min among self-discharge cases, vs. 4 h 25 min (*p* < 0.001) among other cases. Median stay for self-discharge cases was also significantly shorter than the 3 h 47 min among cases medically discharged from the ED (*p* < 0.001). Among the 4723 cases involving opioids, 723 (15.3%) self-discharged after a median stay of 2 h 30 min (IQR 1 h 16–4 h 39 min). The median stay for the 250/723 (34.6%) of these cases given naloxone was 2 h 11 min (IQR 1 h 3–3 h 39 min).

**Table 2:** Self-discharge from the emergency department during acute recreational drug toxicity: demographics and treatment

	**Self-discharge** ***n* (%)**	**Other discharge** ***n* (%)**	***P*-value**
**Males**	1393 (77.1)	10 345 (77.6)	0.63
**Age**^**a,b**^	33 (26–42)	32 (25–40)	< 0.001
**Brought by ambulance**	1282 (70.9)	9378 (70.4)	0.63
**Length of hospital stay**^**a**^	2:20 (1:13–3:59)	4:25 (2:36–7:55)	< 0.001
**Treatment**^**c**^	605 (33.5)	7128 (53.5)	< 0.001
**Intubated**	1 (0.1)	514 (3.9)	< 0.001
**Sedation**	120 (6.6)	2641 (19.8)	< 0.001
**Naloxone**	295 (16.3)	2157 (16.2)	0.91
**Flumazenil**	10 (0.6)	303 (2.3)	< 0.001
**Other antidote**	1 (0.1)	62 (0.5)	0.019
**Total**	1807 (100)	13 328 (100)	

^a^Median (interquartile range)

^b^Age not registered in 135 cases

^c^Any treatment beyond mere observation

**Fig. 1:**
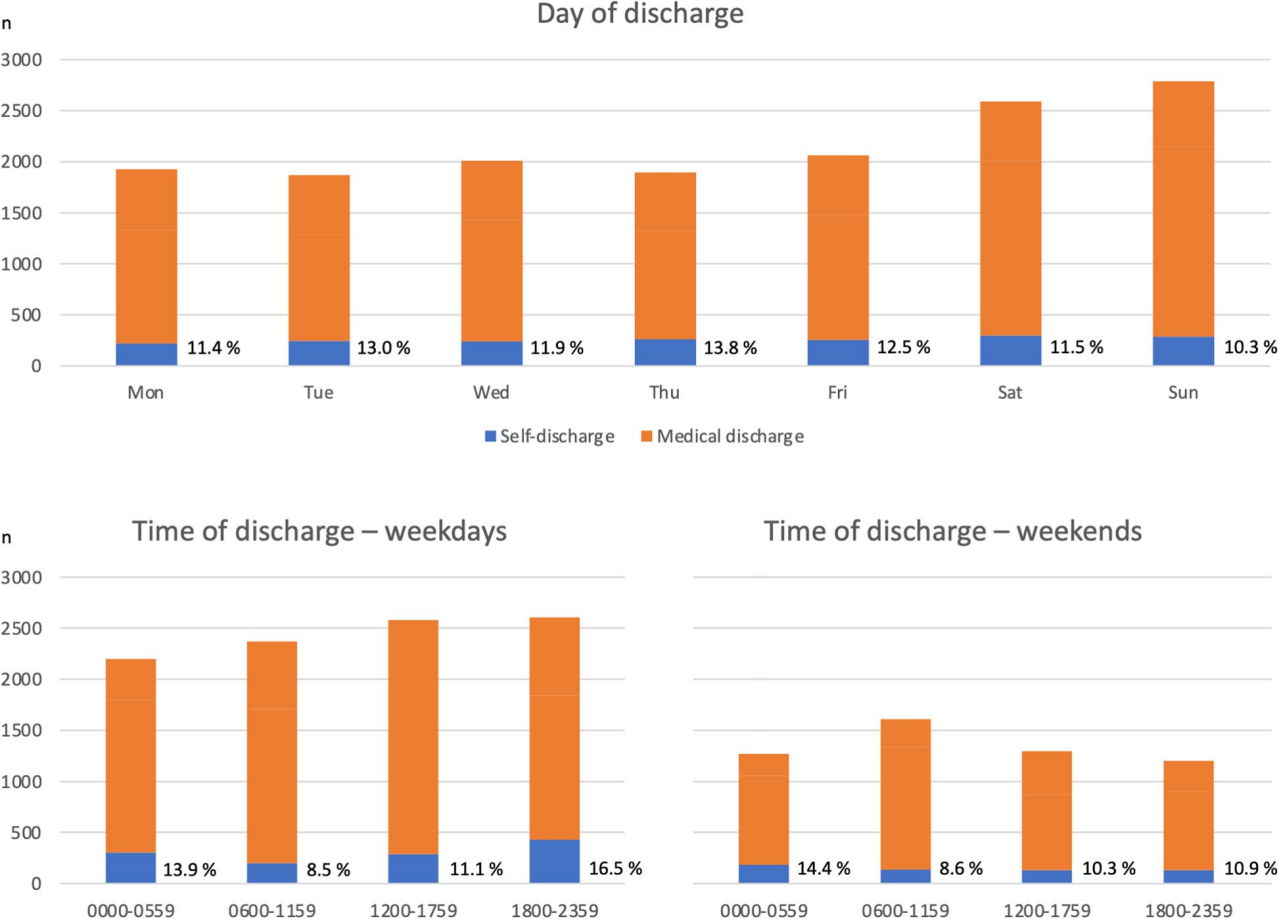
Day and time of discharge from the emergency department

There was a relatively even spread of self-discharge across the days of the week (10.3 to 13.8%) (Fig. 1). Self-discharge rates were highest on weekday evenings (16.5%) and overnight on both weekends (14.4%) and weekdays (13.9%).

Heroin was associated with self-discharge, adjusted odds ratio of 1.44 (95% confidence interval 1.26–1.64), as were synthetic cannabinoids, 1.44 (1.10–1.89) (Table 3). Naloxone was given to 1285 (35.5%) of the 3615 cases who had taken heroin. Among these 1285 cases, 216 (16.8%) self-discharged.

**Table 3:** Drugs and self-discharge from the emergency department during acute recreational drug toxicity

	**Total** ***n* (%)**	**Self-discharge** ***n* (%)**	**Crude OR**	**95% CI**	***P* value**	**Adjusted OR**	**95% CI**	***P* value**
**Heroin**	3615 (23.9)	599 (16.6)	1.70	1.53–1.89	< 0.001	**1.44**	1.26–1.64	< 0.001
**Synthetic cannabinoids**	440 (2.9)	73 (16.6)	1.49	1.15–1.92	0.002	**1.44**	1.10–1.89	0.008
**Buprenorphine**	135 (0.9)	21 (15.6)	1.36	0.85–2.18	0.20	1.25	0.78–2.01	0.35
**Cathinones**^**a**^	148 (1.0)	17 (11.5)	0.96	0.58–1.59	0.86	1.19	0.71–1.99	0.51
**Z-drugs**	94 (0.6)	13 (13.8)	1.19	0.66–2.13	0.57	1.19	0.65–2.15	0.58
**Ketamine**	284 (1.9)	33 (11.6)	0.97	0.67–1.40	0.87	1.14	0.79–1.66	0.48
**Benzodiazepines**	2377 (15.7)	346 (14.6)	1.32	1.16–1.50	< 0.001	1.13	0.99–1.30	0.065
**MDMA**	1189 (7.9)	132 (11.1)	0.92	0.76–1.10	0.35	1.00	0.82–1.22	0.99
**Ethanol**^**b**^	6246 (41.3)	683 (10.9)	0.85	0.77–0.94	0.001	0.94	0.84–1.05	0.25
**Amphetamine**	1991 (13.2)	241 (12.1)	1.02	0.88–1.18	0.81	0.92	0.79–1.07	0.28
**Opioids**^**c**^	752 (5.0)	84 (11.2)	0.92	0.73–1.17	0.51	0.88	0.69–1.13	0.32
**Methadone**	436 (2.9)	48 (11.0)	0.91	0.67–1.23	0.54	0.82	0.61–1.12	0.22
**NPS**^**d**^	116 (0.8)	11 (9.5)	0.77	0.41–1.44	0.41	0.81	0.43–1.53	0.52
**Cocaine**	2440 (16.1)	243 (10.0)	0.79	0.68–0.91	0.001	**0.80**	0.68–0.93	0.004
**Cannabis**	2362 (15.6)	233 (9.9)	0.78	0.67–0.90	0.001	**0.79**	0.67–0.92	0.003
**LSD**	198 (1.3)	18 (9.1)	0.74	0.45–1.20	0.22	0.77	0.47–1.25	0.29
**GHB**	2764 (18.3)	246 (8.9)	0.68	0.59–0.78	< 0.001	**0.76**	0.64–0.89	0.001
**Crack**	373 (2.5)	38 (10.2)	0.83	0.59–1.17	0.29	**0.66**	0.47–0.93	0.019
**Methamphetamine**	861 (5.7)	61 (7.1)	0.55	0.42–0.71	< 0.001	**0.65**	0.49–0.85	0.002
**Pregabalin**	99 (0.7)	7 (7.1)	0.56	0.26–1.21	0.14	0.48	0.22–1.04	0.064
**Mephedrone**	404 (2.7)	17 (4.2)	0.32	0.20–0.52	< 0.001	**0.41**	0.25–0.67	< 0.001
**Other**	700 (4.6)	66 (9.4)	0.76	0.59–0.98	0.036	0.82	0.63–1.07	0.14
**Unknown**	898 (5.9)	105 (11.7)	0.98	0.79–1.20	0.81	0.94	0.75–1.18	0.56
**Total**	15,135 (100)	1807 (11.9)						

Logistic regression analysis. Numbers and percentages in the columns add up to more than the totals, as more than one toxic agent was taken in 9796/15135 (64.7%) cases

All drugs were included in the multivariate analysis. Reference groups are cases not involving the specified drug

Odds ratios are adjusted for the variables in the table. Adjusted odds ratios for associations with *p* < 0.05 are given in bold types

*CI* Confidence interval, *GHB* Gammahydroxybutyrate, *LSD* Lysergic acid diethylamide, *MDMA* Methylenedioxymethamphetamine, *NPS* Novel psychoactive substances, *OR* Odds ratio

^a^Cathinones other than mephedrone

^b^Isolated alcohol intoxications not included

^c^Unspecified opioids and opioids other than heroin, methadone, and buprenorphine

^d^Unspecified NPS and NPS other than cathinones and synthetic cannabinoids

The only clinical feature associated with self-discharge was agitation, adjusted odds ratio of 1.27 (1.10–1.46) (Table 4). The presence of nearly any other clinical feature was associated with a lower risk of self-discharge. Naloxone treatment was also associated with self-discharge, 1.27 (1.07–1.51), while sedation, 0.38 (0.30–0.48), and flumazenil treatment, 0.38 (0.18–0.83), were associated with less risk of self-discharging.

**Table 4:** Clinical features and self-discharge from the emergency department during acute recreational drug toxicity

	**Total** ***n* (%)**	**Self-discharge** ***n* (%)**	**Crude OR**	**95% CI**	***P* value**	**Adjusted OR**	**95% CI**	***P* value**
**Tachypnoea ***(RR* > *20/min)*^*a*^	1581 (10.4)	134 (8.5)	0.66	0.55–0.79	< 0.001	**0.79**	0.63–1.00	0.046
**Bradycardia ***(HR* < *50/min)*^*a*^	278 (1.8)	19 (6.8)	0.54	0.34–0.86	0.009	0.62	0.34–1.13	0.12
**Hypertension ***(SBP* ≥ *180 mmHg)*	432 (2.9)	31 (7.2)	0.56	0.39–0.81	0.002	1.00	0.66–1.51	0.98
**Hypotension ***(SBP* ≤ *90 mmHg)*	708 (4.7)	48 (6.8)	0.52	0.39–0.71	< 0.001	**0.60**	0.42–0.86	0.005
**Hyperthermia ***(temp* ≥ *39.0 °C)*	161 (1.1)	3 (1.9)	0.14	0.04–0.44	0.001	**0.20**	0.05–0.83	0.026
**Arrhythmias**	156 (1.0)	4 (2.6)	0.19	0.07–0.52	0.001	0.26	0.06–1.08	0.064
**Palpitations**	1029 (6.8)	64 (6.2)	0.47	0.36–0.61	< 0.001	**0.57**	0.41–0.77	< 0.001
**Chest pain**	969 (6.4)	67 (6.9)	0.53	0.41–0.68	< 0.001	**0.70**	0.52–0.94	0.016
**Vomiting**	1446 (9.6)	106 (7.3)	0.56	0.46–0.68	< 0.001	**0.68**	0.55–0.86	< 0.001
**Headache**	681 (4.5)	58 (8.5)	0.68	0.51–0.89	0.005	0.84	0.62–1.13	0.24
**Psychosis**	897 (5.9)	51 (5.7)	0.43	0.32–0.57	< 0.001	**0.45**	0.31–0.64	< 0.001
**Hallucinations**	1028 (6.8)	72 (7.0)	0.54	0.42–0.69	< 0.001	**0.73**	0.54–0.98	0.034
**Agitation**	3766 (24.9)	445 (11.8)	0.99	0.88–1.10	0.79	**1.27**	1.10–1.46	< 0.001
**Anxiety**	2722 (18.0)	181 (6.6)	0.47	0.40–0.55	< 0.001	**0.64**	0.52–0.77	< 0.001
**Cerebellar features**	196 (1.3)	14 (7.1)	0.56	0.33–0.97	0.040	0.68	0.37–1.24	0.21
**Lowest level of consciousness**								
*Alert (GCS 15)*	4592 (39.5)	618 (13.5)	1			1		
*Drowsy (GCS 9–14)*	4857 (41.8)	619 (12.7)	0.94	0.83–1.06	0.30	**0.66**	0.58–0.76	< 0.001
*Coma (GCS 3–8)*	2166 (18.6)	140 (6.5)	0.44	0.37–0.54	< 0.001	**0.37**	0.30–0.46	< 0.001
**Treatment – sedation**	2761 (18.2)	120 (4.3)	0.29	0.24–0.35	< 0.001	**0.38**	0.30–0.48	< 0.001
**Treatment – naloxone**	2452 (16.2)	295 (12.0)	1.01	0.89–1.15	0.88	**1.27**	1.07–1.51	0.005
**Treatment – flumazenil**	313 (2.1)	10 (3.2)	0.24	0.12–0.45	< 0.001	**0.38**	0.18–0.83	0.015
**Female sex**	3396 (22.4)	414 (12.2)	1.03	0.92–1.16	0.61	1.08	0.94–1.24	0.29
**Age ***20–39 years*	982 (6.5)	64 (6.5)	1			1		
≤ *19 years*	9967 (66.4)	1171 (11.7)	0.52	0.40–0.68	< 0.001	**0.55**	0.40–0.74	< 0.001
≥ *40 years*	4051 (27.0)	549 (13.6)	1.18	1.06–1.31	0.003	1.06	0.93–1.20	0.42
**Total**	15,135 (100)	1807 (11.9)						

*CI* Confidence interval, *GCS* Glasgow coma scale score, *HR* Heart rate, *OR* Odds ratio, *RR* Respiratory rate, *SBP* Systolic blood pressure

Logistic regression analysis. Reference groups for categorical variables are cases without the specified clinical feature, specified treatment not given, or male sex

Odds ratios are adjusted for the variables in the table. Adjusted odds ratios for associations with *p* < 0.05 are given in bold types. Bradypnoea (RR < 12/min), tachycardia (HR ≥ 100/min), and seizures were not included in the multivariate model as both crude and adjusted ORs for these variables were not significantly associated with self-discharge (*p* < 0.05)

Missing: lowest level of consciousness not registered in 3520 cases (total *n* = 11,615, self-discharge *n* = 1377); age not registered in 135 cases

^a^At presentation

## Discussion

### Summary of main findings

Among presentations with acute recreational drug toxicity, 11.9% self-discharged from the ED. Self-discharge rates varied between centres, from 1.7 to 17.1%. In self-discharge cases the patient stayed shorter, 2 h 20 min vs. 4 h 25 min, and less frequently received treatment beyond mere observation, 33.5 vs. 53.5%. Having taken synthetic cannabinoids and heroin was associated with self-discharge, as were agitation and naloxone treatment. Sedation treatment, flumazenil treatment, and the presence of nearly any clinical feature but agitation were associated with a lower risk of self-discharge.

### Self-discharge rates

The overall self-discharge rate of 11.9% was somewhat lower than the 15–19% previously reported among patients treated for recreational drug toxicity [5, 10, 11], but clearly higher than the 1–3% reported in general hospital populations [1–3, 5], and also higher than the 6–11% reported among patients with acute poisoning in general [12–14]. Considering the increased risk of premature death associated with drug overdose [15, 16] and with self-discharge [1, 2], our study substantiates previous findings that patients self-discharging during treatment for recreational drug overdose are at-risk patients in an at-risk situation [10, 11].

The variation of self-discharge rates across the centres in our study may to some extent reflect different discharge procedures and practices. Though self-discharge in part may be related to patient characteristics, situational factors also are at work [1, 21, 22]. We found higher self-discharge rates overnight and on weekday evenings, which might possibly be due to a larger caseload at these times combined with less staff on night shifts. Except for the two Oslo centres, all our data were collected from regular hospital EDs. In Oslo, the OAEOC is a primary care unit functioning as a pre-ED for the city’s hospitals. The less complicated cases are treated there. Hence, the hospitalized cases treated at the OUH are more complicated, and nearly 90% are admitted to intensive care [23]. This intra-city division of tasks may partly explain the high and low discharge rates at the Oslo centres. Another observation worth exploring is that centres with large volumes of presentations tended to have high self-discharge rates. Still, there are several exceptions to this trend, and even more so when looking at the total number of presentations and not just recreational drug toxicity presentations. Hence, other mechanisms than caseload are probably also at work. Some centres require the patient to sign a form before self-discharge, and others do not. At some centres, self-discharge may not be officially allowed, which would probably impact on how potential self-discharge is dealt with. There may also be architectonical differences making the way out of the ED more or less cumbersome. Differences in discharge procedures should be further investigated to find measures to reduce self-discharge.

Specific interventions to reduce the risk of self-discharge have been tried. In a US study of alcohol-intoxicated ED patients, a protocol for identifying and monitoring incapacitated patients and making it more cumbersome for them to leave until they had regained the capacity to make medical decisions, reduced self-discharge from 15.0 to 7.4%, and increased ED stay by 42 min [24]. The incapacitated patients were placed in a well-supervised area without easy egress, their shoes were removed, and their own clothes were replaced with a gown.

Delay in diagnostic and therapeutic procedures is a frequently stated reason for self-discharge [25]. Waiting is tiresome. Hence, striving for rapid medical clearance makes sense as a measure to reduce self-discharge by reducing time spent waiting for procedures or results. On the other hand, what patients perceive as waiting time, clinicians may perceive as observation time necessary to ensure that the risk of harmful toxicity has passed [26].

Not surprisingly, in self-discharge cases, the patient stayed for a shorter time in the ED. They also to a lesser extent received treatment beyond mere observation. Some patients may have self-discharged before recommended treatment or observation, which would put them at risk. Still, in our clinical experience, important treatment for patients with acute recreational drug toxicity (e.g., naloxone for respiratory depression from opioid overdose and sedation for agitated patients) will often be given early after presentation as this is when the patient is most severely affected. Unfortunately, we were not able to explore this in our data set as the time of giving specific treatments was not registered. Furthermore, many patients treated for recreational drug toxicity would benefit from referral to follow-up for substance use disorders, mental health issues, and somatic co-morbidity [27, 28]. Self-discharge often closes these options. On the other hand, some self-discharging patients may have been less severely sick, not in need of any specific treatment, and consequently preferred to self-discharge rather than wait for medical clearance.

### Characteristics of self-discharge

Clinical features of more severe toxicity—hyperthermia, tachypnoea, hypotension, chest pain, arrhythmias, and psychosis—were associated with a reduced risk of self-discharge. The presence of physical symptoms may increase the patient’s concern. Also, clinicians may be more concerned about patients with physical symptoms and therefore put in more effort to prevent them from leaving. In addition, these patients are more likely to be offered treatment which may reduce the risk of self-discharge, e.g., sedation. Still, though only a handful of cases with hyperthermia or arrhythmias self-discharged, substantial numbers with other serious clinical features did.

Naloxone treatment was associated with self-discharge. Though we did not collect information on opioid withdrawal as such, it is possible that naloxone precipitated opioid withdrawal symptoms in some cases leading to self-discharge. This may also partly explain the association between self-discharge and heroin. The median length of stay for self-discharge cases with opioid toxicity receiving naloxone was 2 h 11 min, with one out of four leaving after less than 1 h 3 min. Hence, in many cases, patients with opioid overdose self-discharged sooner than the recommended 2 h of observation after naloxone administration [26]. This may increase their risk of recurrent opioid toxicity, especially in the setting of long-acting opioids, an increasing problem in several European countries [29]. These patients should be targeted for interventions to reduce their risk of self-discharge. Caution should be taken when administering naloxone so as to avoid precipitating withdrawal symptoms. In one out of four cases with opioid overdose, the patient self-discharged after 4 h 39 min, which is long enough for withdrawal symptoms to develop even without naloxone administration. This may also be part of the explanation of the association between self-discharge and heroin. Accordingly, improved management of opioid withdrawal might also reduce self-discharge.

For synthetic cannabinoids, a possible explanation for the association with self-discharge may be agitation, a frequently reported feature of synthetic cannabinoid toxicity [30]. Agitation may also be associated with self-discharge as a feature of opioid withdrawal. Furthermore, it is likely that clinicians let agitated patients leave before medical clearance to reduce risk to staff and as an alternative to sedation treatment. Hence, measures to improve the management of agitated patients may be a fruitful approach in reducing self-discharge, e.g., providing a calm environment, effective and consistent communication, and timely sedation treatment [31–33].

Psychosis, hallucinations, and anxiety were associated with a lower risk of self-discharge, in contrast to agitation. Again, these symptoms may increase the patient’s concern. Healthcare workers may also be more concerned about these patients, hence keeping them on for treatment or observation and sometimes initiating mandatory admission to a psychiatric ward.

### Limitations

The drug(s) involved in the presentations were based on patient self-report and the clinical assessment of the doctor treating the patient. This results in some uncertainty as to which drug(s) were taken. However, toxicologic laboratory testing often confirms the drugs reported by the patient and their companions, though frequently more drugs are found on testing than were reported [34]. Still, this is generally the mainstay of the clinical diagnosis with laboratory testing undertaken in only a minority of presentations in most settings.

As we only registered presentations and not individual patients, we were not able to identify whether any patients re-presented, and hence could not assess the risk of unfortunate outcomes for the self-discharging patients. Furthermore, as self-discharging patients tend to present with repeated poisonings [10], the frequency of characteristics associated with self-discharge may be exaggerated as we have counted them for presentations and not patients.

We did not collect information on discharge procedures at the participating centres. Hence, we were not able to explore the possible procedural mechanisms explaining the large variation in self-discharge rates across the centres.

The data material is six years old. However, as far as we know, interventions to reduce the risk of self-discharge have not been broadly implemented since then. Furthermore, we are not aware of changes in procedures that might affect our results, apart from temporary changes during the recent pandemic.

## Conclusion

The self-discharge rate of 11.9% among presentations with acute recreational drug toxicity is high, suggesting, for many patients, a higher risk of further episodes as well as missed opportunities for treatment and for referral to treatment. There was a large variation in self-discharge rates across the participating centres, possibly partly reflecting different discharge procedures and practices. Further research on different discharge procedures and targeted interventions may provide information on useful approaches to reduce self-discharge. Heroin, synthetic cannabinoids, and agitation increased the risk of self-discharge, as did naloxone treatment. Rapid medical clearance, improved management of agitated patients, and careful administration of naloxone to avoid precipitating opioid withdrawal symptoms may be approaches worth exploring.

## Data Availability

The datasets generated and analysed during the current study are not publicly available as several manuscripts based on Euro-DEN Plus data are in preparation, but are available from the corresponding author on reasonable request.
